# Fecal Microbiota Alterations Associated With Diarrhea-Predominant Irritable Bowel Syndrome

**DOI:** 10.3389/fmicb.2018.01600

**Published:** 2018-07-25

**Authors:** Xiaojun Zhuang, Zhenyi Tian, Li Li, Zhirong Zeng, Minhu Chen, Lishou Xiong

**Affiliations:** Department of Gastroenterology and Hepatology, The First Affiliated Hospital of Sun Yat-sen University, Guangzhou, China

**Keywords:** irritable bowel syndrome, small intestinal bacterial overgrowth, gut microbiota, rifaximin, 16S rRNA gene-targeted pyrosequencing

## Abstract

Altered gut microbiota are assumed to be involved in the pathogenesis of irritable bowel syndrome (IBS). However, gut microbiota alterations reported in different studies are divergent and sometimes even contradictory. To better elucidate the relationship between altered gut microbiota and IBS, we characterized fecal microbiota of diarrhea-predominant IBS (IBS-D) patients and further explored the effect of rifaximin on gut microbiota using bacterial 16S rRNA gene-targeted pyrosequencing. In our study, IBS-D patients defined by Rome III criteria and age-and-gender matched healthy controls (HC) were enrolled to investigate the fecal microbiota alterations. These IBS-D patients were then treated with rifaximin for 2 weeks and followed up for 10 weeks. Fecal microbiota alterations, small intestine bacterial overgrowth (SIBO) and gastrointestinal (GI) symptoms of IBS-D patients were analyzed before and after treatment. Our results showed fecal microbiota richness but not diversity was decreased in IBS-D patients as compared to HC and there were alterations of fecal microbiota at different taxonomy levels. The abundant phyla *Firmicutes* was significantly decreased and *Bacteroidetes* was increased in IBS-D patients. Moreover, the alterations of predominant fermenting bacteria such as *Bacteroidales* and *Clostridiales* might be involved in the pathophysiology of IBS-D. In addition, rifaximin was effective in terms of SIBO eradication and even GI symptoms of IBS-D patients achieved at least 10-week improvement after treatment. Furthermore, rifaximin induced alterations of some special bacteria rather than affected the overall composition of microbiota in IBS-D patients. Meanwhile, a potential decrease in propanoate and butanoate metabolism was found in these IBS-D patients after rifaximin treatment. Taken together, there were alterations of gut microbiota in IBS-D patients as compared to HC. Rifaximin could relieve GI symptoms, modify gut microbiota in IBS-D patients and eradicate SIBO in those patients with SIBO, suggesting an additional therapeutic mechanism of rifaximin in the treatment of IBS-D. Our findings of compositional gut microbiota alterations in IBS-D and the effect of rifaximin on the gut microbiota implied that altered gut microbiota were associated with the pathogenesis of IBS.

## Introduction

Irritable bowel syndrome (IBS) is one of the most common functional bowel disorders. The prevalence of IBS around the world is 7–21% and it is 1–16% in China, but it differs depending on regions and diagnostic criterions (Xiong et al., 2004; Chey et al., 2015). Accumulating evidences demonstrated that alterations of gut microbiota might be closely associated with IBS. The mechanisms of altered gut microbiota involved in the pathogenesis of IBS may include altering mucosal permeability, inducing visceral hypersensitivity, activating immune reaction, disturbing gastrointestinal motility and affecting brain-gut axis (Holtmann et al., 2016). In addition, small intestinal overgrowth (SIBO) has been blamed for IBS, but there is also no consensus. The incidence of new onset symptoms following acute infectious gastroenteritis might also suggest a microbiological pathogenesis on IBS.

Disturbances in the composition and stability of gut microbiota with subsequent short chain fatty acids (SCFAs) alterations in IBS patients had been explored using different experimental techniques (Balsari et al., 1982; Noor et al., 2010; Tana et al., 2010; Carroll et al., 2011; Chassard et al., 2012). Recently, high throughput sequencing as a sequencing strategy has been applied to study the gut microbiota of IBS in recent years. For example, Carroll et al. (2012) reported less microbial richness and a higher level of the phylum *Proteobacteria* (particularly the class *γ-Proteobacteria* and the family *Enterobacteriaceae*) in IBS-D patients through 454 Pyrosequencing. Interestingly, our previous meta-analysis concluded that decreased *Bifidobacteria* and *Lactobacillus* and increased *Escherichia Coli* and *Enterobacterium* were found in Chinese IBS patients, while decreased *Bifidobacteria* and increased *Bacteroides* were observed in IBS patients from other regions around the world (Zhuang et al., 2016). In another meta-analysis, down-regulated *Lactobacillus*, *Bifidobacterium* and *F. prausnitzii* were reported particularly in IBS-D patients (Liu et al., 2017).

Manipulating gut microbiota through administration of probiotics, prebiotics, antibiotics (including highly selective agents and non-absorbable varieties), modifications of dietary and fecal microbiota transplantation are promising approaches for IBS. Non-absorbable antibiotics offer the opportunity for gut microbiota modification without the possibility of systematic adverse effects. Two randomized double-blind placebo controlled phase 3 trials (TARGET 1 and TARGET 2) had shown the efficacy of a 2 week course of rifaximin for global IBS symptoms and bloating in IBS-D patients (Pimentel et al., 2011). The possible mechanism of rifaximin management might include altering the production and metabolism of bacteria-produced agents, preserving epithelial function, modulating bacterial virulence and reducing pro-inflammatory cytokine production (Pimentel, 2016). However, some IBS patients do not present visible abnormalities in the microbiota composition after rifaximin intervention (Soldi et al., 2015).

Currently, limited information is available regarding whether rifaximin used in IBS-D patients has an impact on bacterial species in the human gastrointestinal tract. The aim of our study was to characterize the fecal microbiota alterations in Chinese IBS-D patients and to further explore the effect of rifaximin on gut microbiota using 16S rRNA gene-targeted pyrosequencing. We also evaluated the impact of rifaximin treatment on GI symptoms and SIBO eradication in IBS-D patients to further identify the relationship between altered gut microbiota and IBS-D.

## Materials and Methods

### Ethics Statement

This study was conducted at the Department of Gastroenterology and Hepatology, the First Affiliated Hospital of Sun Yat-sen University, from September 2015 to December 2016. The protocol was approved by the Medical Ethical Committee of the First Affiliated Hospital of Sun Yat-sen University and all patients had written informed consent. ClinicalTrials.gov ID: NCT02565654.

### Study Subjects

Thirty IBS-D patients and 13 HCs were recruited into our study by two gastroenterologists with expertise in IBS. All patients enrolled in this study fulfilled Rome III diagnostic criteria for IBS and aged between 18 and 65 years. Patients were excluded if they had inflammatory bowel disease, history of duodenal or gastric ulcer, diverticulitis or infectious gastroenteritis, previous abdominal surgery, lactose intolerance, metabolic diseases, human immunodeficiency virus infection and renal, cardiac or hepatic disease. Subjects taking any antibiotics, probiotics, prebiotics, corticosteroids, proton-pump inhibitors, or IBS prescription medications 4 weeks prior to baseline of our study were also excluded from our study. Age- and gender- matched HCs had no concomitant diseases, recurring GI symptoms, clinically significant abnormalities and medication taken.

### Study Design and Procedures

Rifaximin (400 mg) were administered orally twice per day for 2 weeks to IBS-D patients (Majewski and McCallum, 2007; Di Stefano et al., 2011). They were further followed up for an additional 10 weeks after treatment. IBS-D patients were asked to fill up a GI symptoms questionnaire at baseline (T0), at the end of the treatment (T14), at the end of the 2-week follow-up (T28), at the end of the 6-week follow-up (T56), and at the end of the 10-week follow-up (T84). The GI symptoms questionnaire including abdominal pain, abdominal discomfort, abdominal distension, diarrhea, defecatory urgency and incomplete evacuation was rated on a 7-point Likert scoring system (with 0 = not at all, 1 = hardly, 2 = somewhat, 3 = moderately, 4 = a good deal, 5 = a great deal, and 6 = a very great deal). Stool samples collected from HCs and IBS-D patients at baseline were used to analyze the characterization of fecal microbiota in IBS-D. Fecal sample collection and lactulose hydrogen breath test (LHBT) were performed at T0 and T28 to observe the effect of rifaximin modification on gut microbiota.

### LHBT

LHBT was performed under standard conditions. Patients did not receive any antibiotics, probiotics, prebiotics, and laxatives in 4 weeks preceding the test. To minimize basal H_2_ excretion, IBS-D patients were asked to avoid foods containing complex carbohydrates (bread, potato, corn) and fiber in the previous evening and required to be fasting for at least 12 h before the breath test. Cigarette smoking and physical exercises were not allowed for 2 h before and during the test. On the day of testing, patients did a mouth wash with 20 mL of 0.05% chlorhexidine (Koutai, Shenzhen, China) to eliminate the fermentation by oropharyngeal bacteria flora. LHBT was performed in IBS-D patients using a gas analyzer (GastroLyzer^®^ Breath Hydrogen Monitor; Bedfont Science Ltd., Britain). Immediately before the test, a sample of expired air was taken to assay the basal H_2_ concentrations, then 25 g lactose dissolved in 100 ml water was administered within 30 s and the expired air was sampled every 30 min in the next 3 consecutive hours.

According to the literature and our previous study (Wang et al., 2015), LHBT was considered as indicative of the presence of SIBO when: (i) a baseline value of H_2_ ≥ 20ppm, and/or (ii) a > 20 ppm increase in H_2_ over basal values within 90 min of lactulose administration.

The LHBT was repeated in all IBS-D patients at T28 to assess the effect of rifaximin treatment on SIBO eradication.

### Sample Collection and DNA Extraction

The collected fecal sample were frozen immediately at −80°C. Fecal bacterial genomic DNA was extracted from 250 mg feces using PowerFecal^®^ DNA Isolation Kit (MoBio, Carlsbad, CA, United States) according to the manufacturer’s instructions. DNA concentration was quantified by NanoDrop2000 Spectrophotometer (NanoDrop products, Wilmington, DE, United States). The extracted DNA samples were eluted and stored in Tris–HCl buffer, pH 8.0, at −20°C.

### Bacterial 16S rRNA Gene Amplification and Miseq Sequencing

PCR amplification of 16S rRNA gene was performed based on the literature using primers specific for the 338–806 (V3–V4) hypervariable regions. The PCR assays were carried out in triplicate as follows: 20 μl reaction solutions with 2 μl template DNA, 4 μl PCR reaction buffer, 0.8 μl of each primer, 2 μl dNTPs, 0.4 μl FastPfu Polymerase (TransGen Biotech, Beijing, China) and 10 μl ddH_2_O. The PCR conditions were as follows: 95°C for 3 min, followed by 27 cycles of 95°C for 30 s, 55°C for 30 s, 72°C for 45 s, and a final extension of 72°C for 10 min, and the mixture was held at 10°C until it was halted by the user. All PCR products were visualized on agarose gels (2% in TAE buffer) containing ethidium bromide, and purified with a DNA gel extraction kit (Axygen, California, United States). Prior to sequencing, the DNA concentration of each PCR product was determined using a QuantiFluorTM-ST fluorescent quantitative system (Promega, WI, United States) and mixed with the appropriate proportion based on sequencing requirements. PE amplicon libraries were constructed and sequencing was performed using the Illumina Miseq platform (Major Bio-Pharm Technology, Shanghai, China).

### Microbiological Analysis

Before analysis, the original data were filtered and optimized to obtain the valid and trimmed sequences through the use of a Trimmomatic trimmer and the FLASH program. Sets of sequences with at least 97% identified were defined as an OTU (operational taxonomic unit) and chimeric sequences were identified and removed using Usearch^1^. The taxonomy of each 16S rRNA gene sequence was analyzed using RDP Classifier^2^ in QIIME^3^ with a confidence threshold of 70%.

Rarefaction curves were plotted for each sample to determine the abundance of communities and sequencing data of each sample. Alpha-diversity analyses, including community richness parameters (Chao1, Ace, Sobs, Rank–Abundance curves), community diversity parameters (Shannon, Simpson, Shannon–Wiener curves) and a sequencing depth index (Good’s coverage) were calculated using the Mothur software^4^. Beta diversity measurements including Hierarchical clustering tree and principal coordinate analyses (PCoA) based on OTU compositions were determined. Bacterial taxonomic distributions of sample communities were visualized using the R package software. Microbiome features differences between groups were analyzed using Mann–Whitney *U*-test. The characterization of microorganismal features differentiating the fecal microbiota specific to different toxigenic types was performed using linear discriminant analysis (LDA) effect size (LEfSe) method^5^. It emphasizes both statistical significance and biological relevance. With a normalized relative abundance matrix, LEfSe uses the Kruskal–Wallis rank sum test to detect features with significantly different abundances between assigned taxa and performs LDA to estimate the effect size of each feature. A significance alpha of 0.05 and an effect size threshold of 2 were used for all potentially biomarkers discussed in this study (Segata et al., 2011). PICRUSt (Phylogenetic Investigation of Communities by Reconstruction of Unobserved States) analysis (KEGG level) was applied to predict the functional profiling of microbial communities based on the 16S rDNA sequences (Langille et al., 2013).

### Statistical Analysis

All statistical analysis were performed using SPSS version 23.0 (SPSS, Inc., Chicago, IL, United States) and Graph Prism version 7.0 (GraphPad software, Inc., La Jolla, CA, United States). Continuous data were analyzed using Student’s *t*-test or Mann–Whitney *U*-test when appropriate. Categorical data were analyzed using Chi-square test. All tests for significance were two-sided and *p* < 0.05 were considered statistically significant.

### Data Availability

The sequences analyzed during this study are available in the SRA database (SRA accession number: SRP150089).

## Results

### Characteristics of the Subjects

Thirty patients with IBS-D (M/F = 21/9, 32.1 ± 8.11 year) and 13 HCs (M/F = 8/5, 30.54 ± 6.75 year) were enrolled in our study. There were no significant difference in gender (χ^2^ = 0.30; *P* = 0.59), age (*t* = 0.61; *P* = 0.55) and body mass index (20.61 ± 3.26 vs. 20.84 ± 1.46, *t* = 0.34; *P* = 0.74) between IBS-D patients and HCs. IBS-D patients and HCs enrolled didn’t take concomitant medications 4 weeks prior to the study and during the follow-up. Stool culture for pathogenic bacteria, yeast, parasites, and viruses were negative in all IBS-D patients and HCs before fecal collection.

### Characterization of Fecal Microbiota in IBS-D Patients

Three IBS-D patients didn’t collect fecal samples at baseline. Accordingly, fecal samples from 27 IBS-D patients and 13 HCs were analyzed using high throughput sequencing to observe the characterization of fecal microbiota in Chinese IBS-D patients at T0. Across the 40 samples, an average of 3,0298 high-quality reads (range from 2,1053 to 3,9523) with a median read length of 471 base pairs were obtained after quality trimming and chimera checking. The comparisons of fecal microbiota richness parameters, diversity parameters and Good’s coverage between IBS-D and HC were summarized in **Table 1**. The values of Good’s coverage were nearly 99.9% for all sequences in these two groups so that sequencing depth were sufficient for investigating fecal microbiota. Both Chao1 and Sobs indices demonstrated that the fecal microbiota richness in IBS-D patients was significantly lower than that in HCs (*P* < 0.05). In contrast to the richness indices, there were no significant differences in the diversity indices (Shannon and Simpson) between IBS-D patients and HCs. To better understand the shared richness among the two groups, a Venn diagram displaying the overlaps between groups was developed. It showed that 322 of the total richness of 427 OTUs were shared among all the samples (Supplementary Figure S1). The Rarefaction, Shannon–Wiener, and Rank-abundance curves generated using R software, as shown in Supplementary Figures S2–S4, were clustered at the 97% phylotype similarity level. Hence, sequencing depth was sufficient for exploring the microbial community of all fecal samples.

**Table 1:** Fecal microbiota community indices between IBS-D patients and HCs.

Community indices	HC (mean)	HC (*SD*)	IBS-D (mean)	IBS-D (*SD*)	*P*-value
Coverage	0.99913	0.00034532	0.9993	0.00024742	0.06238
Ace	162.18	38.824	139.21	34.456	0.05983
Chao1	167.23	41.177	136.72	37.552	0.02188^∗^
Sobs	144.57	36.836	120.59	35.102	0.04816^∗^
Shannon	2.887	0.49344	2.6359	0.49553	0.1315
Simpson	0.13536	0.069777	0.16463	0.066562	0.1966

*SD, Standard Deviation; IBS-D, Diarrhea-predominant Irritable Bowel Syndrome; HC, healthy control. ^∗^P < 0.05 was considered statistically significant.*

**FIGURE 1:**
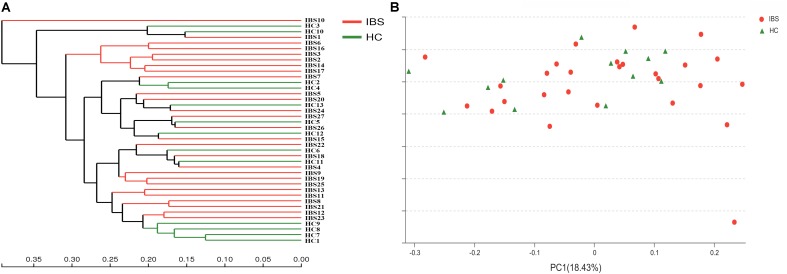
Structural comparison of fecal microbiota between IBS-D patients and HCs. **(A)** Hierarchical clustering analysis of fecal microbiota in IBS-D patients and HCs. Fecal microbiota trees were generated using the UPGMA (unweighted pair group method with arithmetic mean). **(B)** Principal coordinate analysis (PCoA) of fecal microbiota communities in IBS-D patients and HCs based on UniFrac metric. Distances between symbols on the ordination plot reflect relative dissimilarities in community structures. IBS, irritable bowel syndrome; IBS-D, diarrhea-predominant IBS; HC, healthy controls.

To measure the extent of the similarity between microbial communities, we examined the relationship of gut microbiota from these 40 feces samples using Bray–Curtis distances, which were shown by a Hierarchical clustering dendrogram. As shown in **Figure 1A**, each branch on the tree represented one sample. Interestingly, the overall trend was that gut microbiota of some IBS-D patients clustered together firstly (IBS4, IBS8, IBS9, IBS11, IBS12, IBS13, IBS19, IBS21, IBS23, IBS25) and then clustered with HC group. It revealed that fecal microbiota from IBS-D patients and HCs could not be divided into two different clusters completely. On the PCoA plot (**Figure 1B**), each symbol representing one sample is used to examine the community structures of gut microbiotas in these two groups. Similar to the Hierarchical clustering analysis, fecal microbiota from IBS-D patients and HCs could not be separated clearly into two different clusters.

**FIGURE 2:**
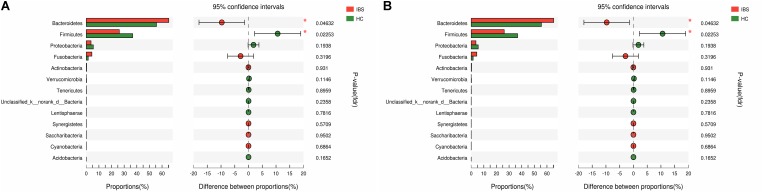
Taxonomic differences of fecal microbiota between IBS-D patients and HCs. **(A)** Comparison of relative abundance at phyla level between IBS-D patients and HCs. **(B)** Comparison of relative abundance at order level between IBS-D patients and HCs. ^∗^Indicates statistically significance (^∗^0.01 < *p* ≤ 0.05, ^∗∗^0.001 < *p* ≤ 0.01). IBS, irritable bowel syndrome; IBS-D, diarrhea-predominant IBS; HC, healthy controls.

**FIGURE 3:**
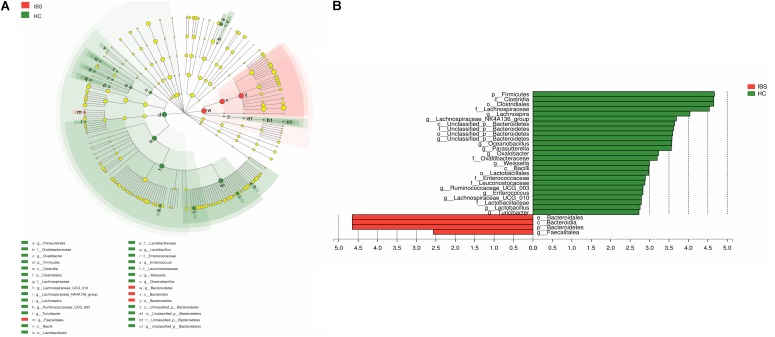
LEfSe identified the most differentially abundant taxons between IBS-D patients and HCs. **(A)** Taxonomic cladogram obtained from LEfSe analysis of 16S sequences. (Red) tax enriched in IBS-D patients; (green) tax enriched in HCS. The brightness of each dot is proportional to its effect size. **(B)** HC-enriched taxa are indicated with a positive LDA score (green) and taxa enriched in IBS-D have a negative score (red). Only taxa meeting an LDA significant threshold ≥2 are shown. IBS, irritable bowel syndrome; IBS-D, diarrhea-predominant IBS; HC, healthy controls.

The taxonomy of fecal microbiota was assessed by a taxon-dependent analysis using the RDP classifier. Thirteen phyla in the HCs and 12 phyla in the IBS-D patients were found in the fecal microbiota of all samples (Supplementary Figure S5). Totally, sequences from all fecal microbiota could be classified into 155 genera, with 141 genera in HCs and 147 genera in IBS-D patients (Supplementary Figure S6). In addition, the comparison in the fecal microbiota at different taxon levels between these two groups was also explored using the Mann-Whitney U test. At the phylum level, *Bacteroidetes* (64.64%), *Firmicutes* (26.14%), *Fusobacteria* (5.18%), and *Proteobacteria* (3.73%) were the most predominant phyla in fecal samples of IBS-D patients, while those in HCs were *Bacteroidetes* (56.43%), *Firmicutes* (35.97%), *Proteobacteria* (5.66%), and *Fusobacteria* (1.39%). As compared to HC, the abundant phyla *Firmicutes* (*P* = 0.046) was significantly decreased and *Bacteroidetes* (*P* = 0.023) was increased in the fecal microbiota of IBS-D patients (**Figure 2A**). At the order level, the abundant orders *Bacteroidales* (*P* = 0.046) was increased in IBS-D patients, while *Clostridiales* (*P* = 0.035) and *Lactobacillales* (*P* = 0.007) were decreased (**Figure 2B**). At the genus level, *Lachnospira* (*P* = 0.036), *Parasutterella* (*P* = 0.033), *Lachnospiraceae_UCG-010* (*P* = 0.026), *Ruminococcaceae_UCG-003* (*P* = 0.023), *Lactobacillus* (*P* = 0.021), *Turicibacter* (*P* = 0.022), *Enterococcus* (*P* = 0.002), *Weissella* (*P* = 0.048), *Oxalobacter* (*P* = 0.048), *Oceanobacillus* (*P* = 0.011), and *Lachnospiraceae_NK4A136_group* (*P* = 0.026) were significantly less abundant in IBS-D patients, while *Faecalitalea* (*P* = 0.012) was increased in IBS-D patients (**Table 2**). To identify the specific bacterial taxa associated with IBS, we compared the fecal microbiota of IBS-D patients and HCs using LEfSe. The results of LEfSe suggested that several bacteria belonging to the *Bacteroidetes* could be used as distinguishing biomarkers for IBS-D (**Figure 3**). Collectively, these characteristic alterations in the fecal microbiota revealed that intestinal dysbiosis is associated with IBS-D.

**Table 2:** Relative abundance of fecal microbiota at order level between IBS-D patients and HCs.

Genus	HC-mean (%)	HC-*SD* (%)	IBS-mean (%)	IBS-*SD* (%)	*P*-value
Lachnospira	4.033	3.867	1.825	2.678	0.03599
Lachnospiraceae_NK4A136_group	1.513	2.337	0.7368	1.903	0.02604
Parasutterella	1.297	2.213	0.7642	1.51	0.03268
Lachnospiraceae_UCG-010	0.2297	0.33	0.1249	0.2094	0.02579
Ruminococcaceae_UCG-003	0.2201	0.3655	0.06357	0.1115	0.02285
Lactobacillus	0.04397	0.04041	0.01706	0.03207	0.02051
Turicibacter	0.043	0.06227	0.007165	0.01681	0.02237
Enterococcus	0.02325	0.0464	0.003341	0.008906	0.002459
Weissella	0.01682	0.04444	0.0005171	0.002121	0.04824
Faecalitalea	0.0009556	0.002654	0.01374	0.02795	0.01233
Oxalobacter	0.006978	0.01498	0.0006981	0.003099	0.04824
g__Unclassified_p__Bacteroidetes	0.005427	0.01451	0	0	0.04278
g__Oceanobacillus	0.0008217	0.001573	0	0	0.01144

*Sd, Standard Deviation; IBS-D, Diarrhea-predominant Irritable Bowel Syndrome; HC, healthy control. P < 0.05 was considered statistically significant.*

### Rifaximin Intervention for SIBO, GI Symptoms, and Fecal Microbiota in IBS-D Patients

**FIGURE 4:**
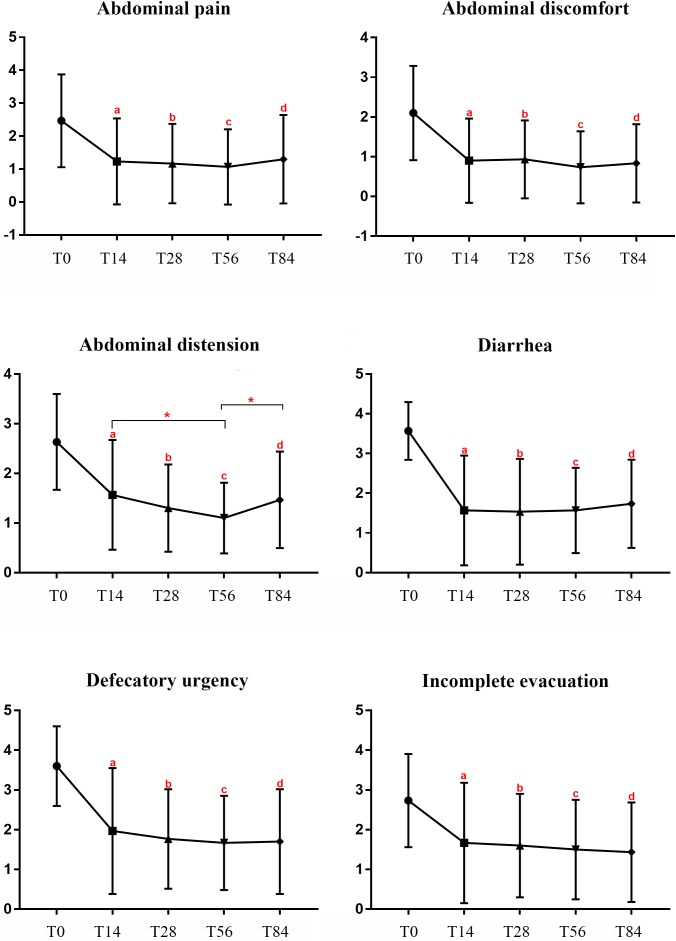
GI symptoms scores in IBS-D patients at different times during the study. Notes: T0: baseline, T14: at the end of 14 days of treatment, T28: at the end of the 2-week follow-up period, T56: at the end of the 6-week follow-up period, T84: at the end of the 10-week follow-up period. ^a^*P* < 0.01:T14 vs. T0, ^b^*P* < 0.01:T28 vs. T0, ^c^*P* < 0.01:T56 vs. T0, ^d^*P* < 0.01:T84 vs. T0, ^∗^*P* < 0.05. IBS, irritable bowel syndrome; IBS-D, diarrhea-predominant IBS. GI symptoms, gastrointestinal symptoms.

At baseline, 14 of 30 IBS-D patients had a diagnosis of SIBO confirmed by LHBT. After 2-week rifaximin treatment, 9 of 14 patients with SIBO (64.29%) had a negative LHBT at T28. Further, the GI symptoms of abdominal pain, abdominal discomfort, abdominal distension, diarrhea, defecatory urgency and incomplete evacuation improved significantly after rifaximin treatment when compared to T0 (*P* < 0.01), and GI symptoms relief maintained at least 10 weeks (**Figure 4**).

**FIGURE 5:**
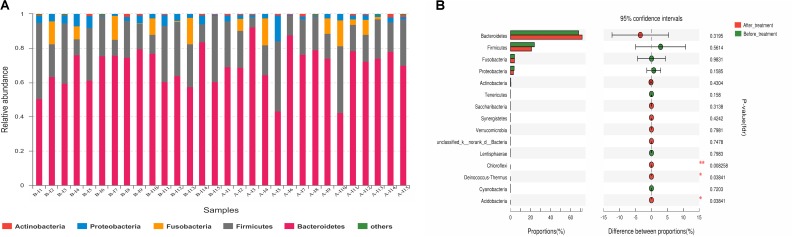
Fecal microbiota in IBS-D patients at the phylum level before and after treatment. **(A)** Relative abundance of fecal microbiota community at the phylum level in IBS-D patients. Less than 1% abundance of the phyla was merged into others. **(B)** Comparison of relative abundance at phyla level in IBS-D patients before and after rifaximin treatment. ^∗^Indicates statistically significance (^∗^0.01 < *p* ≤ 0.05, ^∗∗^0.001 < *p* ≤ 0.01). IBS, irritable bowel syndrome; IBS-D, diarrhea-predominant IBS.

**FIGURE 6:**
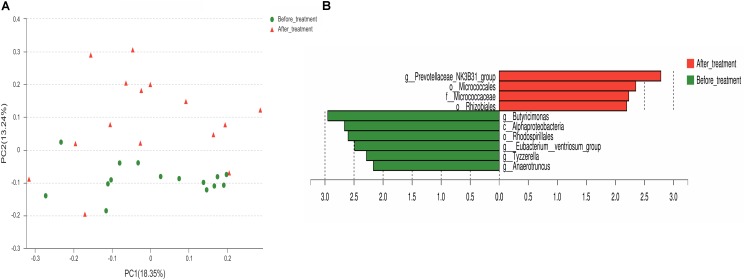
Structures of fecal microbiota in IBS-D patients before and after rifaximin treatment. **(A)** PCoA plot of the fecal microbiota based on the unweighted UniFrac metric. **(B)** The most differentially abundant taxons identified by LEfSe in IBS-D patients before and after rifaximin treatment. Enriched taxa in IBS-D patients after rifaximin treatment are indicated with a positive LDA score (red) and taxa enriched in IBS-D patients before rifaximin treatment have a negative score (green). Only taxa meeting an LDA significant threshold ≥ 2 are shown. IBS, irritable bowel syndrome; IBS-D, diarrhea-predominant IBS.

Fecal samples from 15 IBS-D patients selected randomly were analyzed to explore rifaximin modification for gut microbiota in IBS-D patients at T0 and T28. Across the 30 samples, an average of 3,0844 high-quality reads (range from 2,1153 to 3,9857) with a median read length of 468 base pairs after quality trimming and chimera checking. Totally, 380 OTUs were identified in IBS-D patients at T0 and 419 OTUs were identified at T28 (Supplementary Figure S7). The Rarefaction, Shannon-Wiener and Rank-abundance curves showed sufficient sequencing depth (Supplementary Figures S8–S10). The fecal microbiota richness parameters, diversity parameters and Good’s coverage from each sample were presented in **Table 3**. The values of Good’s coverage were nearly 99.9% for all sequences in the fecal samples so that sequencing depth was sufficient to investigate the fecal microbiota of all samples. Unexpectedly, there were no significant differences in the fecal microbiota richness parameters (Ace, Chao1, and Sobs) and diversity parameters (Shannon and Simpson) in IBS-D patients before and after treatment. However, there were some alterations of the fecal microbiota diversity in IBS-D patients after rifaximin treatment as the comparison of Shannon indices revealed. Of 17 identified phyla, *Bacteroidetes*, *Firmicutes*, *Fusobacteria*, and *Proteobacteria* were predominant phyla (>1% of the total DNA sequences) in all 30 samples (**Figure 5A**). At the phylum level, *Chloroflexi* (*P* = 0.008), *Deinococcus-Thermus* (*P* = 0.038), and *Acidobacteria* (*P* = 0.038) were increased in IBS-D patients after rifaximin treatment (**Figure 5B**). At the class level, *Cytophagia*, *Deinococci*, and *Acidobacteria* were more enriched in IBS-D patients at T28. But *Alphaproteobacteria* was decreased. At the order level, higher levels *Micrococcales*, *Rhizobiales*, *Sphingomonadale*s, *Acidimicrobiales*, *Rhodobacterales*, *Methylophilales*, *Propionibacteriales*, *Frankiales*, *Cytophagales*, *Deinococcales*, *Streptomycetales*, *Nitriliruptorales*, and *Micromonosporales* were detected in IBS-D patients at T28. But the levels of *Rhodospirillales* become lower. At the genus level, detected OTUs were distributed among 226 different bacterial genera in total samples, and there were significant difference in several genus of fecal microbiota between T0 and T28. Among the abundant genus, *Bacillus* (*P* = 0.004), *Kocuria* (*P* = 0.02), *Arthrobacter* (*P* = 0.01), and Devosia (*P* = 0.004) were significantly more abundant and *Butyricimonas* (*P* = 0.02),*Eubacterium ventriosum* (*P* = 0.03)*, Anaerotruncus* (*P* = 0.03), and *Tyzzerella* (*P* = 0.02) were reduced in IBS-D patients after treatment (Supplementary Table S1). PCoA showed that bacterial communities before treatment clustered tightly and separated from those after treatment along principal coordinate axis 2 (PC2) with 15.54% variation. However, there were several samples overlapped (**Figure 6A**). LEfSe analysis suggested that *Prevotella*, *Micrococcus*, and *Rhizobiales* might be distinguishing biomarkers for fecal microbiota of IBS-D patients after rifaximin treatment (**Figure 6B**).

**Table 3:** Fecal microbiota community indices in IBS-D patients before and after rifaximin treatment.

Community indices	Before treatment-mean	Before treatment-*SD*	After treatment-mean	After treatment-*SD*	*P*-value
Coverage	0.99923	0.00028651	0.99911	0.00042233	0.3674
Ace	152.04	39.061	141.13	41.907	0.467
Chao1	151.64	39.497	143.7	42.799	0.6018
Sobs	134.2	34.443	122.33	35.734	0.3624
Shannon	2.6856	0.48063	2.3642	0.40777	0.05819
Simpson	0.16387	0.072524	0.21067	0.098494	0.1495

*SD, Standard Deviation; IBS-D, Diarrhea-predominant Irritable Bowel Syndrome. P < 0.05 was considered statistically significant.*

**FIGURE 7:**
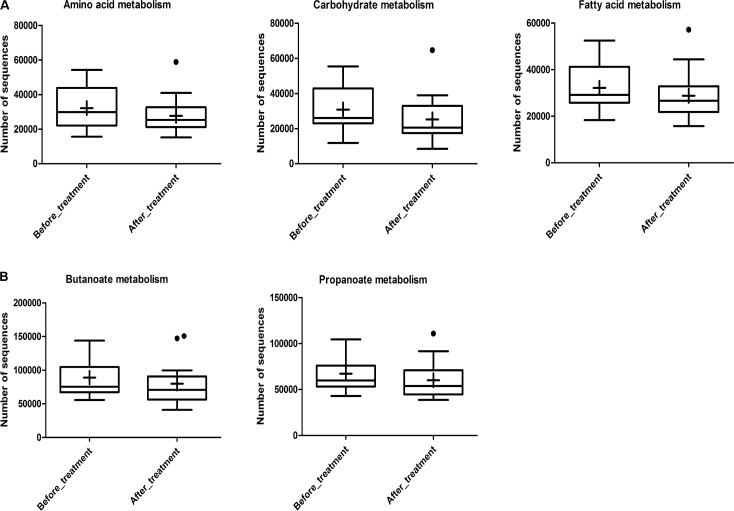
Inferred gut microbiome functions by PICRUSt from 16S rRNA gene sequences in IBS-D patients before and after rifaximin treatment. **(A)** Basic metabolism including amino acid metabolism, carbohydrate metabolism, and fatty acid metabolism. **(B)** SCFA production including propanoate and butanoate metabolism. Box plots denote the top quartile, median and bottom quartile, and the “+” mean the average value as well as the “∙” means the outlier. IBS, irritable bowel syndrome; IBS-D, diarrhea-predominant IBS.

To investigate the gut microbiome functions related to the rifaximin treatment, we adopted PICRUSt to infer putative metagenomes from 16S rRNA gene profiles. The summarized information of PICRUSt analysis was shown in **Figure 7**. The basic metabolism of gut microbiome including amino acid metabolism, carbohydrate metabolism and fatty acid metabolism, were compared in these IBS-D patients before and after rifaximin treatment (**Figure 7A**). However, there were no differences of these basic metabolisms in IBS-D patients before and after treatment. Moreover, a potential decrease of propanoate and butanoate metabolism in the IBS-D patients after rifaximin treatment was found (**Figure 7B**), which possibly due to the reduction of SCFAs-producing bacteria, indicating a possible therapeutic mechanism of rifaximin for IBS-D.

## Discussion

In our study, we found that there were alterations of gut microbiota in Chinese IBS-D patients as compared to those in HC. Further, GI symptoms relief, SIBO normalization and gut microbiota modification after rifaximin treatment observed in our study implied that altered gut microbiota were associated with the pathogenesis of IBS-D.

Despite significant inter-individual variation, a large decrease in *Firmicutes* with concomitant increase in *Bacteroidetes* was observed in Chinese IBS-D patients in our study. However, the results from other studies are inconsistent of an increase, decrease, or no change in *Firmicutes* and *Bacteroidetes* within IBS patients were reported (Rajilic-Stojanovic et al., 2011; Jeffery et al., 2012; Jalanka-Tuovinen et al., 2014). In addition, the percentages of some fermenting bacteria (particularly *Bacteroidales* and *Clostridiales*) were altered in these IBS-D patients. Alteration of SCFAs might be involved in the pathophysiology of IBS by activing the mucosal immune system, increasing intestinal permeability, activing sensory pathways and modulating the enteric motility. In addition, lactic acid-producing bacteria such as *Lactobacillales* were decreased significantly in IBS-D patients, which was similar to the findings from several early studies. The decrease or elimination of lactic acid buildup will impair the intestinal defense barrier and increase osmotic load in the intestinal lumen, finally leading to diarrhea (Rokana et al., 2016). As shown in our results, butyrate-producing bacteria such as *Ruminococcaceae* and *Lachnospiraceae* were also decreased dramatically. Butyrate is a preferred energy source for colonic epithelial cells to maintain normal intestinal barrier function. Overall, there were alterations of gut microbiota at different taxon levels in IBS-D patients as compared to HC and the obvious alterations of SCFA-producing bacteria implied that altered gut microbiota was associated with IBS-D.

One way used to confirm the relationship between altered gut microbiota and IBS is to provide a therapy directed specifically at gut microbiota to achieve symptomatic improvement or even cure (Quigley, 2017). Rifaximin is a gastrointestinal-selective antibiotic that has shown efficacy in IBS patients, and it had been approved by Food and Drug Administration for the treatment of IBS-D in 2015. In our study, a short course of rifaximin could lead to sustained improvement of GI symptoms in IBS-D patients, normalize SIBO in 64.29% of IBS-D patients with SIBO, and the plausible mechanism of rifaximin effect might be modifying some bacterial groups.

Evidence from other studies found that rifaximin altered the composition of bacterial communities including *Lactobacillus* and *Bifidobacterium* species in animal experiments. For human, findings from Soldi et al. (2015) and Zeber-Lubecka et al. (2016) found that rifaximin did not induce dramatic alterations of gut microbiota in IBS patients, but fluctuations in few bacterial groups. In our study, rifaximin treatment modified some bacterial groups rather than altered the overall composition of the core microbiota in IBS-D patients. The results from taxonomic composition and community structures showed that there were no significant differences of the predominant phylum *Bacteroidetes*, *Firmicutes*, *Fusobacteria*, and *Proteobacteria* in IBS-D patients at T28 when compared to those at T0, but *Chloroflexi*, *Deinococcus-Thermus*, and *Acidobacteria* were increased in IBS-D patients after rifaximin treatment. Unlike the abundant *Bacteroidetes* and *Firmicutes* phylum in the human gut, *Chloroflexi*, *Deinococcus-Thermus.* and *Acidobacteria* were scarcely studied and explored, limiting us to analyze the specific role of these bacteria involved in IBS. At the genus level, 28 genus existed significant differences in IBS-D patients between T0 and T28. Decreased *Butyricimonas*, *Eubacterium ventriosum*,*Eubacterium oxidoreducens*, *Anaerotruncus*, *Tyzzerella*, *Peptococcus*, and increased *Kocuria*, *Bacillus, Arthrobacter*, *Nocardioides*, *Pontibacter*, *Streptomyces*, *Rhizobium*, *Nitriliruptor*, *Asanoa*, and *Prevotella* were detected in IBS-D patients at T28, suggesting that presumed mechanism of rifaximin might be modifying gut microbiota for IBS-D patients. The decreased butyrate-mediating bacteria were presented in the IBS-D patients after rifaximin treatment, such as *Butyricimonas* and *Eubacterium ventriosum*, which might explain the potential decrease of butanoate metabolism. It meant there was a reduction in the butyrate concentration in IBS-D patients after rifaximin treatment, which was consistent with the reports from Acosta et al. (2016). Interestingly, findings from our study were also in agreement with those reported by Maccaferri et al. (2010) and Brown et al. (2010) who concluded that potential mechanisms of rifaximin include a direct bactericidal modification with pathogen adhesion reduction and inflammatory processes regulation. Consistent with previous studies (Gatta and Scarpignato, 2017), our study also found that rifaximin intervention was effective for the normalization of SIBO. Due to the small sample size, it was impossible to speculate the differences of fecal microbiota between IBS-D patients with SIBO and without SIBO. The efficacy of SIBO eradication might be explained by the effect of rifaximin on gut microbiota.

There are several limitations in our study. Firstly, the sample size is small so that the conclusion should be drawn cautiously. In addition, we did not analyze the relationship between GI symptoms relief and SIBO eradication. Thirdly, the effects of rifaximin on metabolite level should be further explored.

## Conclusion

In summary, we explored characterization and rifaximin modification for fecal microbiota in Chinese IBS-D patients with high throughput sequencing in this study. Alterations of butyrate-producing and lactic acid-producing bacteria might contribute to the symptoms of IBS-D. Rifaximin could relieve GI symptoms in IBS-D patients and normalize SIBO in 64.29% of IBS-D patients with SIBO. Meanwhile, rifaximin intervention could induce alterations of some special bacteria rather than dramatic shifts in overall composition of gut microbiota. However, there were no differences in amino acid metabolism, carbohydrate metabolism and fatty acid metabolism. The potential decrease in propanoate and butanoate metabolism in the IBS-D patients after treatment might be a possible therapeutic mechanism of rifaximin. The precise mechanisms of rifaximin effect are not fully understood and further studies are warranted to define characteristic candidate bacteria or metabolites relevant to IBS and provide evidences of mechanism about the effect of probiotics and antibiotics on IBS patients.

## Author Contributions

XZ designed and performed the study, analyzed the results, and drafted the manuscript. LX designed the study, analyzed the results, and edited the manuscript. XZ and ZT performed sample collection and lactose hydrogen breath test (LHBT). LL administered clinical data recording. ZZ and MC contributed to strategic development decisions and clinical supervision. All authors approved the final version of the manuscript.

## Conflict of Interest Statement

The authors declare that the research was conducted in the absence of any commercial or financial relationships that could be construed as a potential conflict of interest.

## Footnotes

1 http://drive5.com/uparse/

2 http://rdp.cme.msu.edu/

3 http://qiime.org/scripts/assign_taxonomy.html

4 https://www.mothur.org/

5 http://huttenhower.sph.harvard.edu/galaxy/
