# Supplementary material for: Fecal Microbiota Alterations Associated With Diarrhea-Predominant Irritable Bowel Syndrome
Source: Front Microbiol. 2018 Jul 25;9:1600. doi: 10.3389/fmicb.2018.01600 (PMC6068233; doi:10.3389/fmicb.2018.01600)
Supplement: Supplementary file 1 [file Image_1.PDF]

SUPPLEMENTARY MATERIAL

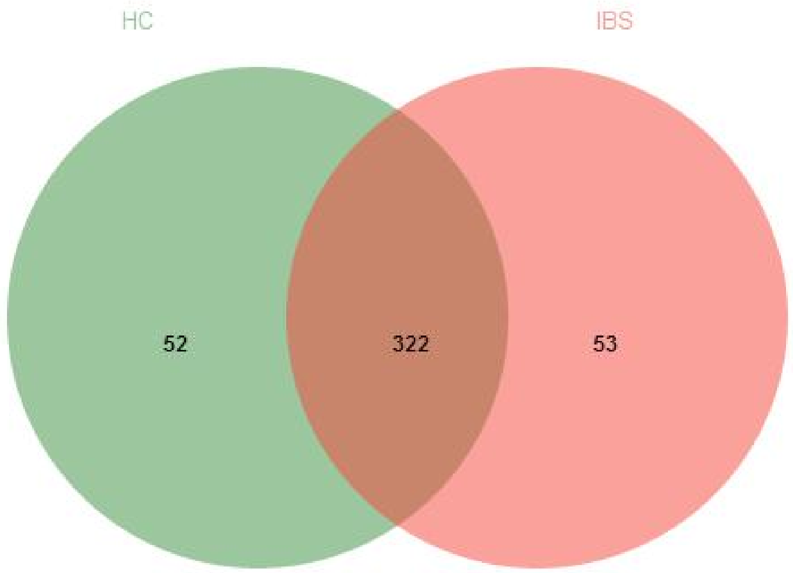

Figures S1 | Venn diagram illustrating overlap of OTUs in fecal microbiota between IBS-D patients and HCs.

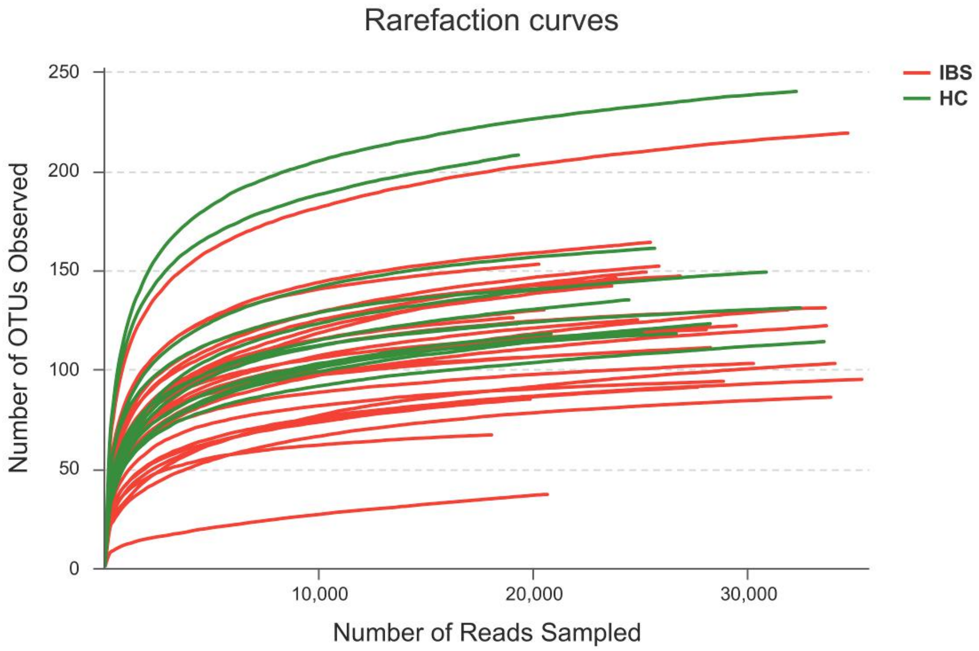

Figures S2 | Rarefaction curves of 40 samples from IBS-D patients and HCs.

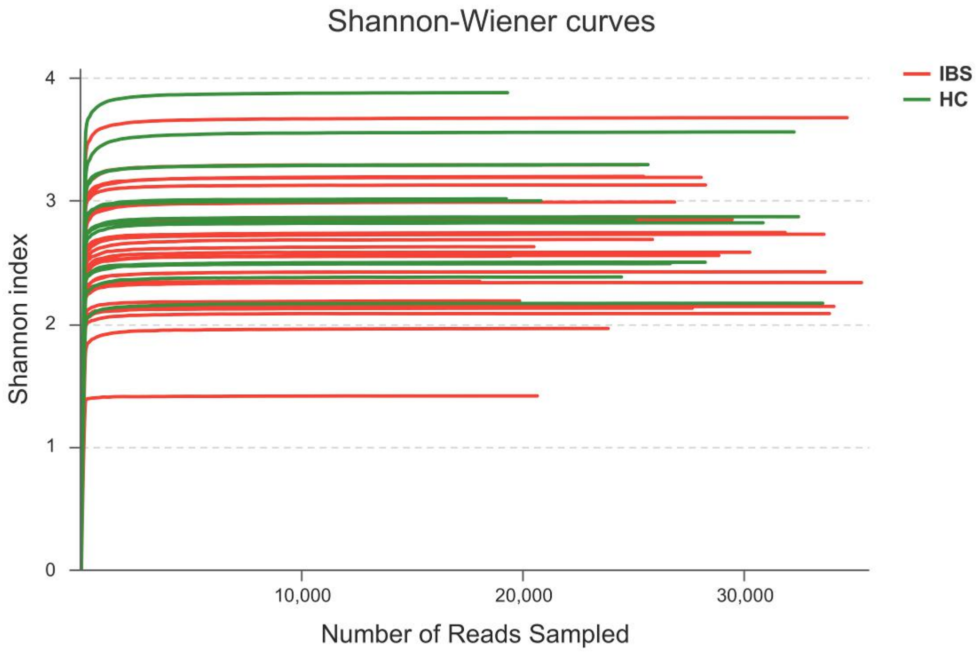

Figures S3｜Shannon–Wiener curves of 40 samples from IBS-D patients and HCs.

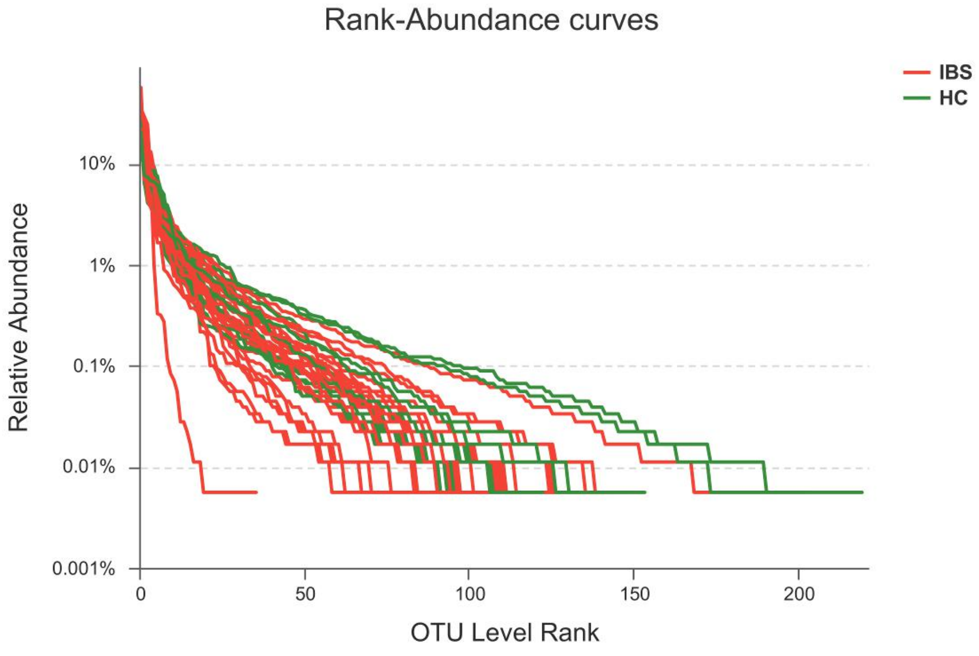

Figures S4｜Rank-abundance curves of 40 samples from IBS-D patients and HCs.

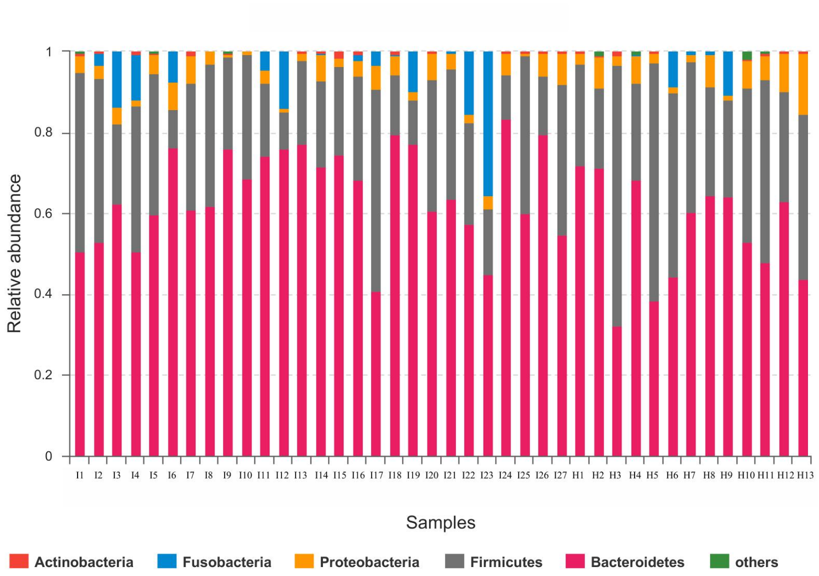

Figures S5丨Relative abundance of fecal microbiota community at the phylum levels from IBS-D patients and HCs.

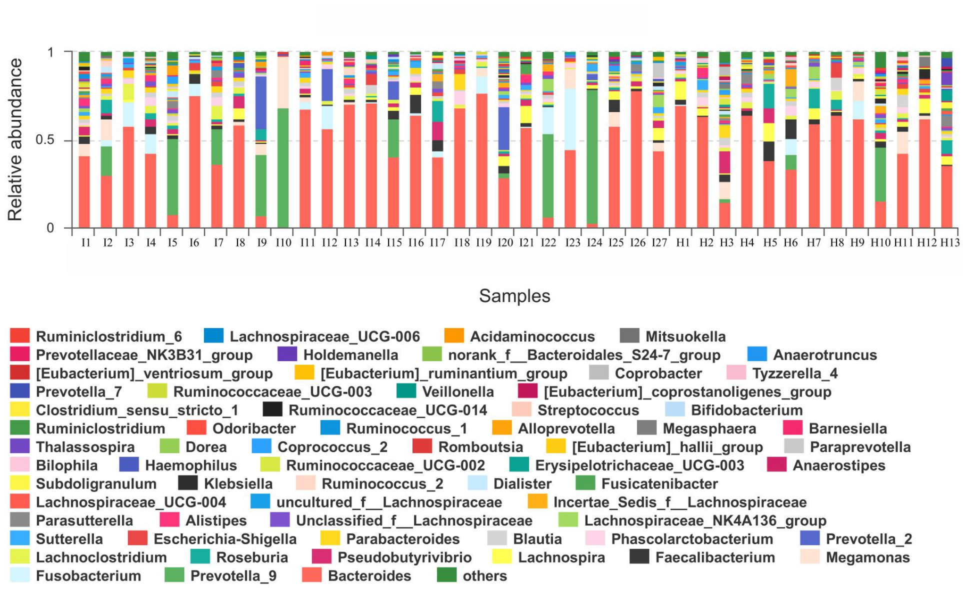

Figures S6丨Relative abundance of fecal microbiota community at the genus levels from IBS-D patients and HCs.

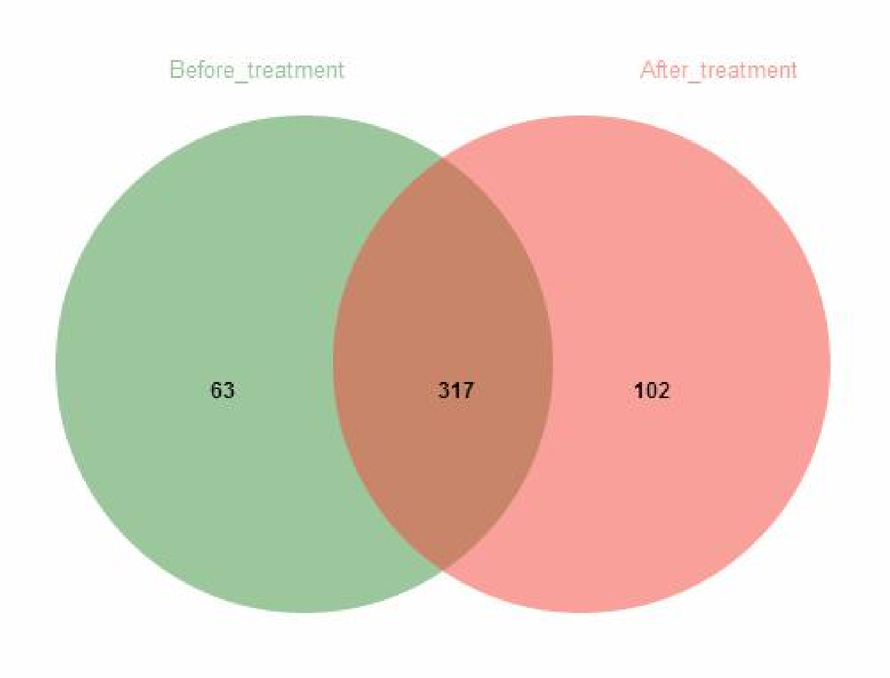

Figures S7 | Venn diagram illustrating overlap of OTUs in fecal microbiota from IBS-D patients before and after rifaximin treatment.

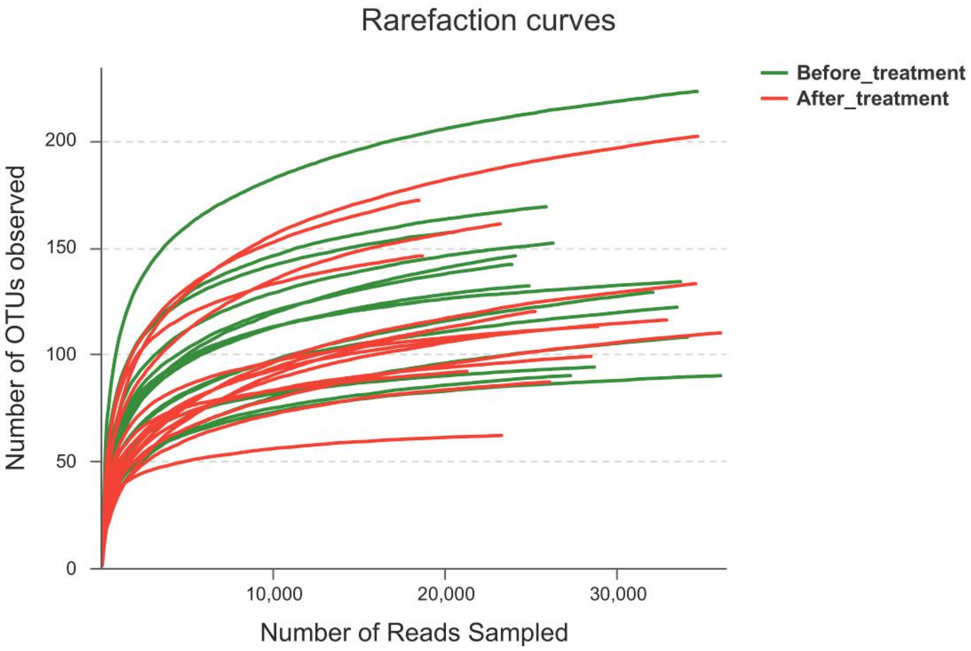

Figures S8 | Rarefaction curves of 30 samples from IBS-D patients before and after rifaximin

treatment.

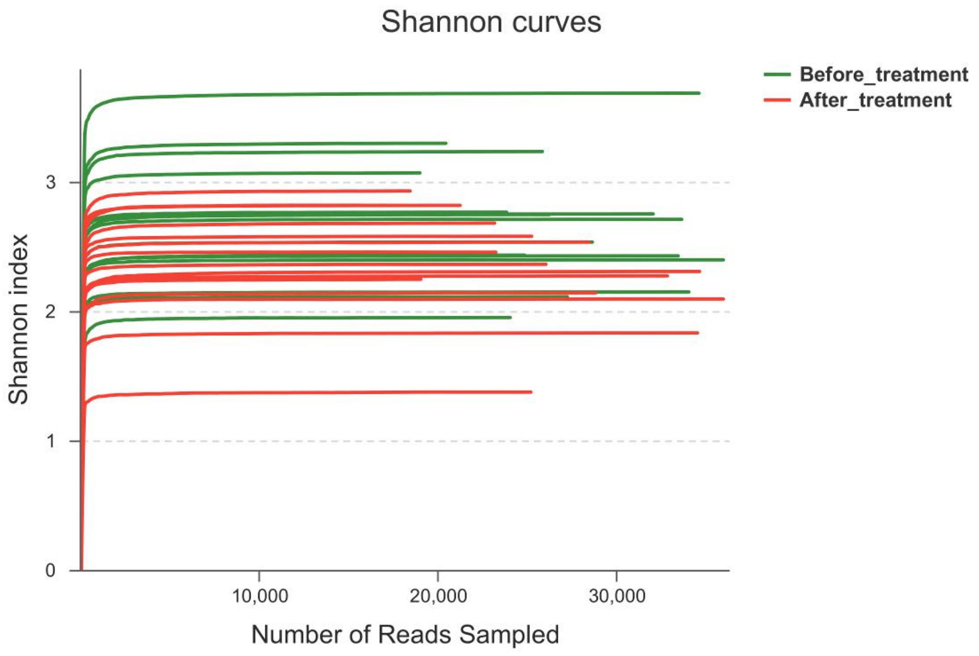

Figures S9 | Shannon–Wiener curves of 30 samples from IBS-D patients before and after rifaximin treatment.

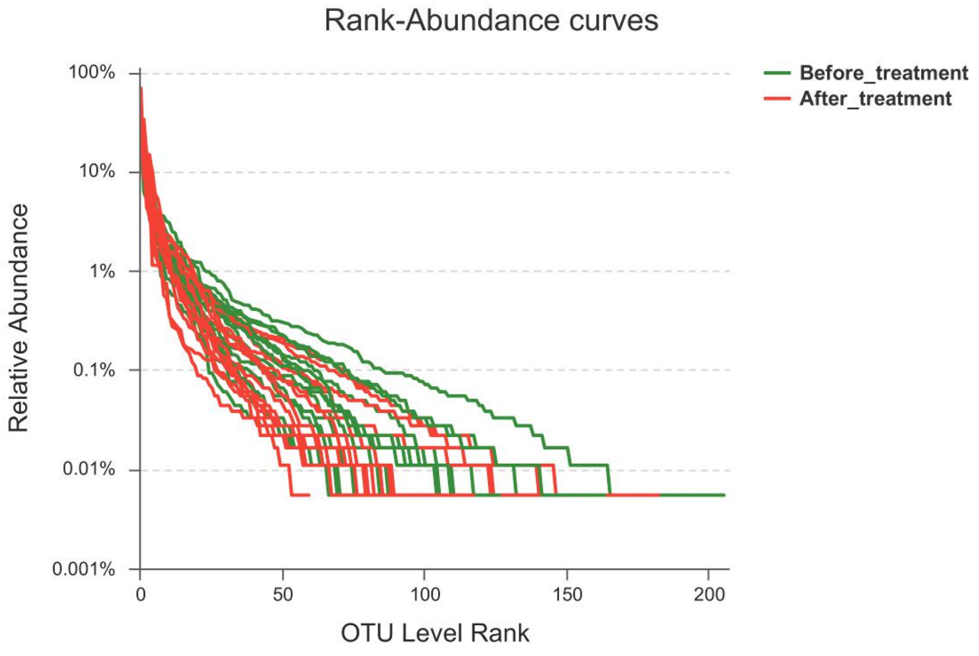

Figures S10 | Rank-abundance curves of 30 samples in IBS-D patients before and after rifaximin treatment.

Table S1 | Comparison of relative abundance at phyla level in IBS-D patients before and after rifaximin treatment

Species name	After_treatment-Mean(%)	After_treatment-Sd(%)	Before_treatment-Mean(%)	Before_treatment-Sd(%)	*P* value
g__Butyricimonas	0.07366	0.1186	0.2303	0.285	0.03106
g__[Eubacterium]_ventriosum_group	0.01525	0.02511	0.08013	0.09842	0.03205
g__Anaerotruncus	0.02946	0.07705	0.05502	0.1074	0.00893
g__Tyzzerella	0.007077	0.02741	0.03807	0.09504	0.02196
g__Kocuria	0.01611	0.04067	0	0	0.0181
g__Arthrobacter	0.01405	0.02549	0.0002822	0.001093	0.01227
g__Family_XIII_UCG-001	0.001085	0.004203	0.01086	0.0252	0.04401
g__norank_f__Bacillaceae	0.01123	0.0328	0	0	0.03841
g__[Eubacterium]_oxidoreducens_group	0.0019	0.002217	0.008289	0.008392	0.034
g__Devosia	0.007135	0.00843	0.0005183	0.002007	0.004173
g__uncultured_o__Frankiales	0.007264	0.01552	0	0	0.003625
g__norank_f__MNC12	0.007128	0.01666	0	0	0.0181
g__unclassified_f__Erythrobacteraceae	0.007076	0.009417	0	0	0.008258
g__Sphingomonas	0.006631	0.01148	0.000209	0.0008096	0.004899
g__uncultured_o__Acidimicrobiales	0.006554	0.01058	0	0	0.008258
g__unclassified_f__Phyllobacteriaceae	0.006158	0.007919	0	0	0.001523
g__Truepera	0.005932	0.01087	0	0	0.03841
g__Nocardioides	0.005762	0.01162	0	0	0.008258
g__Peptococcus	0	0	0.005605	0.01073	0.03841
g__unclassified_f__Microbacteriaceae	0.005537	0.01097	0	0	0.0181
g__Pontibacter	0.004462	0.006643	0	0	0.008258
g__Streptomyces	0.003947	0.006464	0.0002591	0.001004	0.01227
g__Rhizobium	0.003846	0.009024	0	0	0.03841

g__Nitriliruptor	0.003648	0.005289	0	0	0.003625
g__norank_c__Gitt-GS-136	0.003574	0.008006	0	0	0.0181
g__norank_o__Subgroup_6	0.002741	0.00574	0	0	0.03841
g__Prevotellaceae_UCG-001	0.002343	0.006207	0	0	0.03841
g__Asanoa	0.001548	0.002668	0	0	0.0181
